# StreetScouting dataset: A Street-Level Image dataset for finetuning and applying custom object detectors for urban feature detection

**DOI:** 10.1016/j.dib.2023.109042

**Published:** 2023-03-08

**Authors:** Sotirios Moschos, Polychronis Charitidis, Stavros Doropoulos, Anastasios Avramis, Stavros Vologiannidis

**Affiliations:** a DataScouting, 30 Vakchou Street, 54629 Thessaloniki, Greece; b Department of Computer, Informatics and Telecommunications Engineering, International Hellenic University, Terma Magnisias, 62124 Serres, Greece

**Keywords:** Street Data, Urban objects, Object detection, Deep learning

## Abstract

The recent advancements in the field of deep learning have fundamentally altered the manner in which certain challenges and problems are addressed. One area that stands to greatly benefit from such innovations is the realm of urban planning, where the utilization of these tools can facilitate the automatic detection of landscape objects in a given area. However, it must be noted that these data-driven methodologies necessitate significant amounts of training data to attain desired results. This challenge can be mitigated through the application of transfer learning techniques, which reduce the amount of required data and permit the customization of these models through fine-tuning. The present study presents street-level imagery, which can be utilized for fine-tuning and deployment of custom object detectors in urban environments.

The dataset comprises 763 images, each accompanied by bounding box annotations for five landscape object classes, including trees, waste bins, recycling bins, shop storefronts, and lighting poles. Furthermore, the dataset includes sequential frame data obtained from a camera mounted on a vehicle, capturing a total of three hours of driving, encompassing various regions within the city center of Thessaloniki.

**Specifications Table**

**Table:** 

Subject	Computer Science
Specific subject area	Computer Vision and Pattern Recognition
Type of data	Images Annotations GPS data
How the data were acquired	In order to capture visual content and the associated GPS information, the Insta360 Pro2 camera is utilized. The aforementioned camera is a device equipped with six ultra-wide-angle lenses, which allow for the recording of 360° scenes with a coverage angle of 200° and a resolution of 3840x2160 for each lens. To enhance the accuracy of GPS data received by the camera's internal GPS unit, an external antenna has been placed on the GPS socket of the camera. For the purpose of real-time camera control, an antenna has been connected to the RF socket of the wireless access point, and a user-friendly application for survey devices has been employed. This application provides a seamless and convenient means of operating the camera, ensuring the acquisition of high-quality data.
Data format	Raw
Description of data collection	The camera has been mounted on a vehicle using a suction cup base, affixed to the roof of the car. An application specifically designed for wireless remote sensing and real-time camera control has been employed, enabling the specification of both the number of frames per second and the frame resolution size. To ensure the acquisition of unique and relevant footage, precise routes have been carefully planned and implemented beforehand.
Data source location	City/Town/Region: Thessaloniki Country: Greece Latitude and longitude: GPS coordinates are provided as JSON files for each route
Data accessibility	Repository name: Zenodo Data identification number: 10.5281/zenodo.7564876 Direct URL to data: https://doi.org/10.5281/zenodo.7564876
Related research article	Charitidis, P., Moschos, S., Pipertzis, A., Theologou, I. J., Michailidis, M., Doropoulos, S., ... & Vologiannidis, S. (2022). StreetScouting: A Deep Learning Platform for Automatic Detection and Geotagging of Urban Features from Street-Level Images. Applied Sciences, 13(1), 266.

## Value of the Data

• This dataset presents an opportunity for further research and experimentation, allowing for the development and refinement of custom detectors capable of automatically detecting urban objects.
• The aforementioned detectors can be applied to real-life street data, enabling the assessment of their efficacy in real-world settings.
• Urban planning administrators, policymakers, municipal offices, geoinformatics companies and other commercial organizations can benefit from the use of this dataset and the generated machine learning models.
• This dataset also enables experimentation with tracking approaches through the utilization of sequential street frames.
• The dataset has been anonymized, with all human faces and vehicle license plates being blurred.
• The data set is comprised of high-rate data acquisition from a 360° camera, providing a valuable resource in an area where most methods in the bibliography rely on Google Street View data and are limited by license restrictions in their use for urban feature measurement.

## 1 Objective

The dataset described in this paper was generated in the context of the GRUBLES project, a research initiative focused on extracting geotagged landscape features in urban areas through the application of machine learning methods. The project's primary objective was to perform a statistical study, aimed at uncovering correlations between landscape features and socioeconomic indicators in a given region. To achieve this end, the deep learning platform “StreetScouting" [1] was developed, providing the means for automatic detection and geotagging of various urban features, including trees, waste bins, recycling bins, shop storefronts, and lighting poles, among others. The annotated portion of the generated dataset was utilized to fine-tune object detectors capable of detecting such features. Street level data from driving routes were also submitted to StreetScouting, allowing for the utilization of various detection and tracking methods to extract valuable information and urban insights for the region of interest.

## 2 Data Description

The dataset consists of two folders. The first folder is named “annotated dataset” and contains the annotated street images. It consists of the folder “images” that has 763 image files. The image format is PNG. 432 images have dimensions of 1080 x 2160 and 331 have dimensions of 866 x 2400. The filenames are random UUIDs. The “annotated dataset” folder also contains the annotations in the file “coco_annotations.json”. Annotations are provided in COCO format [2]. Table 1 shows the total number of annotated objects per class.

**Table 1:** Total number of annotated objects per class.

Class	Annotated Objects
Tree	1922
Waste Bin	223
Recycling Bin	181
Lighting Pole	716
Shop Storefront	628

The second folder is named “routes” and contains consecutive frames of four different driving routes in the city of Thessaloniki and their corresponding GPS signal. So the folder “routes” contains 4 folders in the following format “VID_≤YYYYMMDD≥_≤HHmmSS≥” where Y denotes digits for year, M denotes digits for month, D denotes digits for day, H denotes digits for hour, m denotes digits for minutes and S denotes digits for seconds. Not the filename represents the start of the collection sequence. All street data was collected in 2022. Each route folder has the “images” folder, which contains the consecutive street image data. Image data in this folder is in JPEG format. Each filename in ‘images’ has the frame_≤id≥.jpg format, where id denotes the order of the frame. Table 2 shows more details regarding the number of frames and frame dimension of the driving routes. The total time of driving routes is approximately 3 hours.

**Table 2:** Total number frames and frame dimensions for each of the routes.

Route Name	Frames Number	Frame Dimension	Route duration
VID_20220617_111456	41,650	1080 x 2160	1h 9m 26s
VID_20220210_112926	23.035	866 x 2400	38m, 26s
VID_20220209_114831	18.000	1080 x 2160	30m, 3s
VID_20220209_123323	18.273	1080 x 2160	30m, 30s

**Fig. 1:**
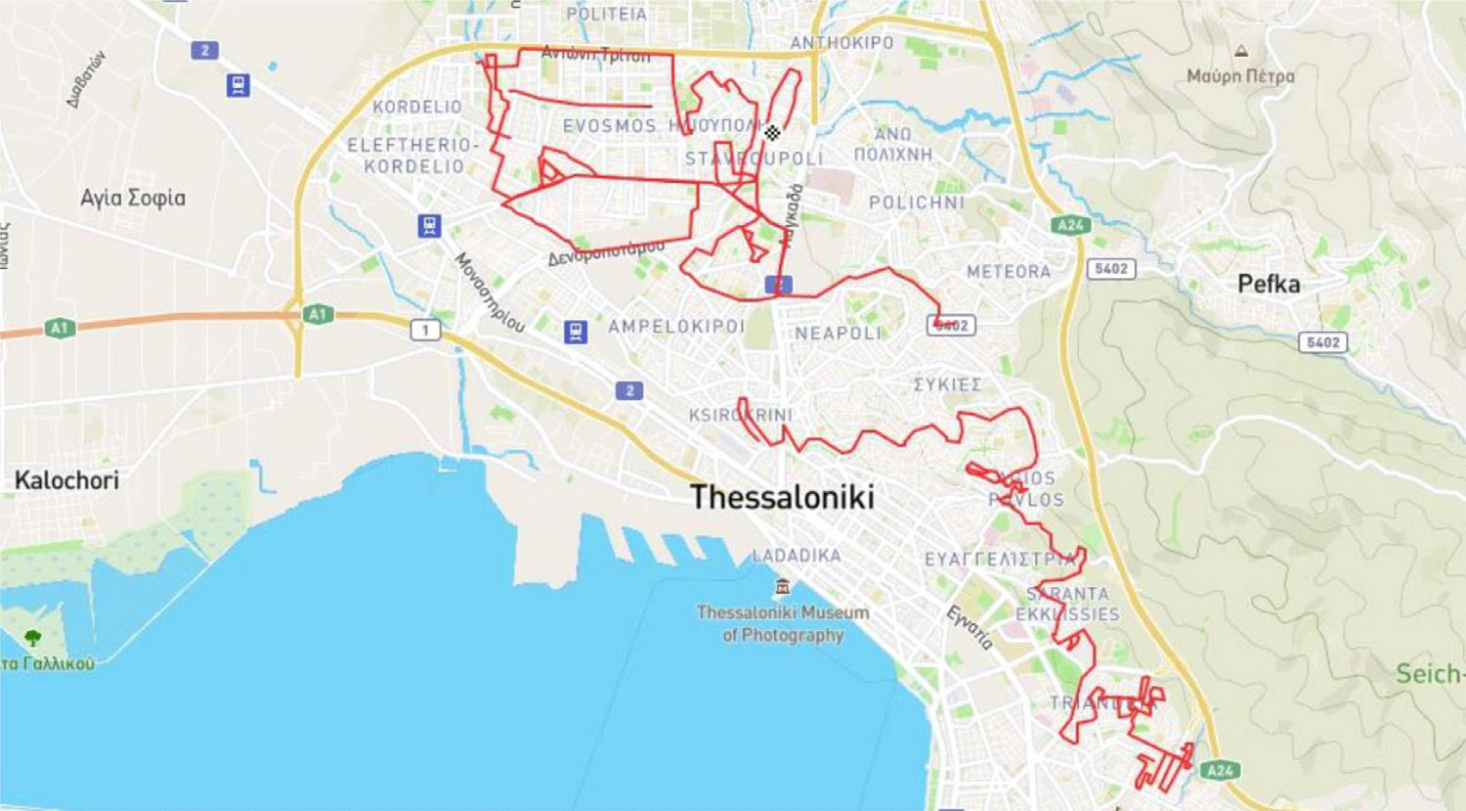
The driving routes of the collected dataset in the city of Thessaloniki.

Each route folder contains a “gps.json” file which contains latitude and longitude information for each frame. This file is essentially a JSON list of objects that each object contains the “frame_name” attribute and the corresponding “coordinates” object which contains the “latitude” and “longitude” attributes. Fig. 1 shows the GPS coordinates of the four routes in a map.

The raw data are publicly available on Zenodo [3].

**Fig. 2:**
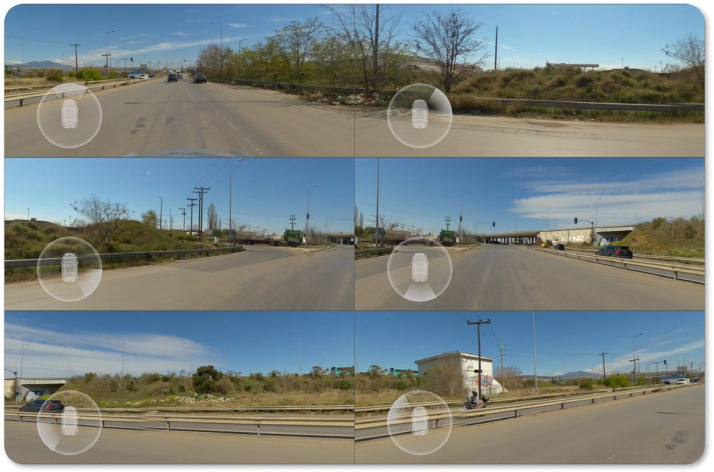
POV of the 6 ultra-wide-angle lenses of Insta360 Pro2.

On the Data Collection subsection of the Experimental Design, Material and Methods section, Fig. 2 illustrates the Point Of View (POV) of the 6 ultra-wide-angle lenses of Insta360 Pro2, where each individual camera has a visual coverage angle of 200°.

**Fig. 3:**
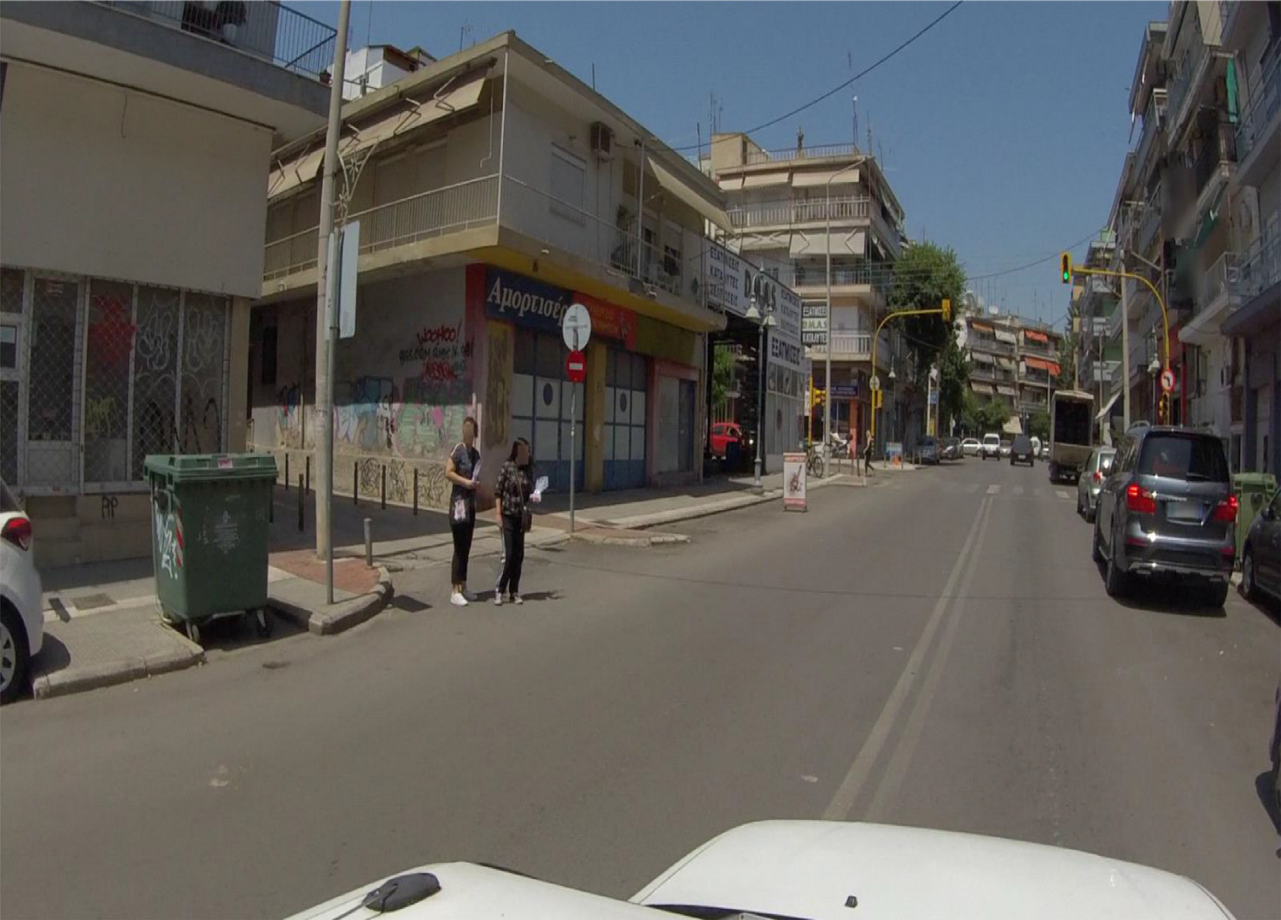
Detection and blurring of bypassing pedestrians and license plates.

On the Depersonalization subsection of the Experimental Design, Material and Methods section, Fig. 3 manifests the proccedings applied to each and every frame of the dataset in order to comply with the GDPR regulations. The blurring of the pedestrian faces and the license plates of the bypassing vehicles deemed essential, as depicted in Fig. 3.

**Fig. 4:**
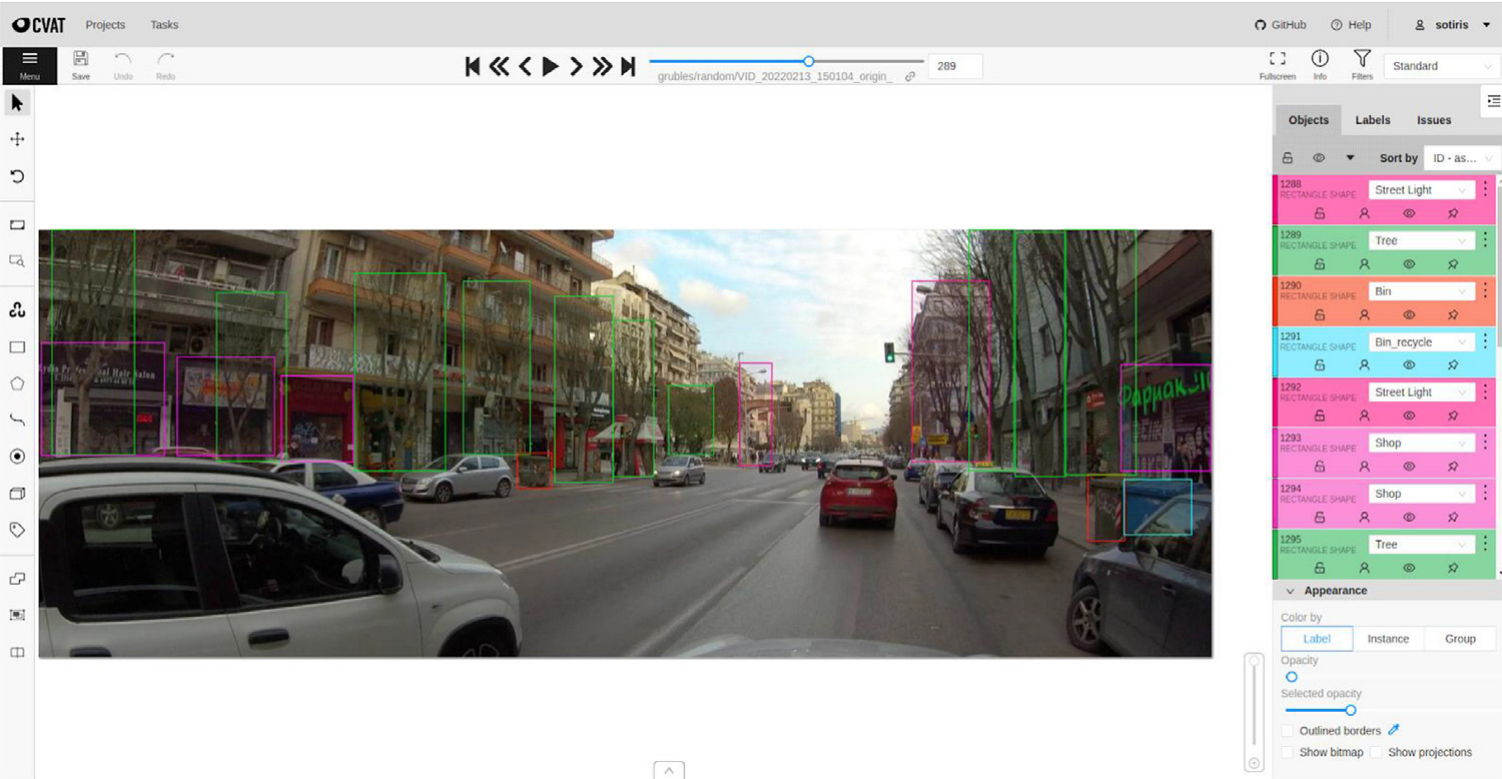
Labeling process snapshot using CVAT.

On the Annotation subsection of the Experimental Design, Material and Methods section, Fig. 4 shows a snapshot of the dataset's labeling process using CVAT.

## 3 Experimental Design, Materials and Methods

### 3.1 Data collection

The dataset was collected utilizing the Insta360 Pro2 camera, which features six ultra-wide-angle lenses, as depicted in Fig. 2. To ensure wireless remote sensing, real-time camera control, and effective wireless pairing, an antenna was connected to the RF socket of the wireless access point and a user-friendly application for survey devices was employed. The application offers customizable options to control the number of frames per second, the frame resolution size, and the real-time stitching of the panoramic view. Despite the short-range of the wireless local connection between the survey device and the camera, the internal GPS unit of the camera may experience signal discontinuities, leading to undefined or missing GPS coordinates. To address this issue, an external antenna was attached to the GPS socket of the camera. The maximum suitable speed for dataset collection, which was determined empirically to avoid GPS distortions, was set at 50 km/h, which is commonly the speed limit in urban areas.

### 3.2 Preprocessing

The footage selected for analysis was extracted from one of the six cameras of the Insta360 Pro2 device, specifically the lens that was positioned towards the front road. The preprocessing of the multimedia stream involved several sequential steps, commencing with the extraction and processing of frames. The frames were selected at a rate of 10 frames per second and their dimensions were adjusted to ensure that the image quality was not impacted by the use of an ultra-wide lens. It is worth noting that there were slight variations in the frame dimensions among certain routes, which were the result of camera settings being altered during the project. The subsequent step was the extraction of GPS information from the metadata of the video, followed by the synchronization of the GPS data with the frames. Any missing data was then filled through linear interpolation.

### 3.3 Depersonalization

In accordance with the provisions of the European General Data Protection Regulation (GDPR), the dataset must strictly adhere to regulations relating to the handling of sensitive data. This requires the detection and obfuscation of both license plates of parked and moving vehicles, as well as the faces of passing pedestrians. To achieve this objective, a deep learning model that has undergone prior training is utilized for the detection of pedestrian faces, followed by the application of a smoothing filter for the purpose of blurring the detected faces. Similarly, a deep learning model that has been fine-tuned for the specific task of license plate detection and blurring is employed, along with the implementation of the aforementioned smoothing filter. A visual representation of a frame that has undergone depersonalization can be found in Fig. 3.

### 3.4 Annotation

To secure a representative portion of the annotated dataset, as outlined in the Data Description subsection, a random sample of 1000 frames was selected. The objects of interest were then subjected to manual annotation, including those which were not present in commonly accessible open-source datasets. The objects annotated included trees, trash bins, recycling bins, street lighting poles, and shops, resulting in a total of 5,228 instances of these objects being annotated, as detailed in Table 2. The annotation process was carried out using the CVAT annotation tool, as depicted in Fig. 4.

## Ethics Statements

This study does not involve experiments on humans or animals. Furthermore, the collected dataset is compliant with Greek's parliament Law 4624/2019: Personal Data Protection Authority, implementing measures of Regulation (EU) 2016/679 of the European Parliament and of the Council of 27 April 2016 on the protection of natural individuals against data processing. More specifically, the General Data Protection Regulation (GDPR) is a comprehensive data privacy regulation enacted by the European Union (EU) to ensure that individuals have control over their personal data and is applied to all organizations operating within the EU and also to organizations outside of the EU if they process personal data of EU residents. One aspect of the GDPR requires that organizations take appropriate measures to protect personal data. In a street-scene dataset collection context, personal data such as faces of pedestrians and license plate numbers are considered to be highly sensitive and can be used to identify individuals. Thus, the blurring of faces and license plates of these individuals is necessary to comply with the GDPR as it minimizes the risk of the personal data being misused or disclosed without consent. By blurring the faces and license plates, the personal data of the individuals is no longer considered to be identifiable and thus, the risk of a data breach or violation of privacy is significantly reduced. By taking this measure, we should can avoid potential fines and legal repercussions while also maintaining the trust and confidence of the individuals whose personal data is being processed.

## CRediT authorship contribution statement

**Sotirios Moschos:** Methodology, Software, Validation, Formal analysis, Investigation, Writing – original draft, Data curation. **Polychronis Charitidis:** Conceptualization, Methodology, Software, Validation, Formal analysis, Investigation, Writing – original draft, Data curation. **Stavros Doropoulos:** Conceptualization, Writing – review & editing, Project administration. **Anastasios Avramis:** Conceptualization, Data curation, Project administration, Funding acquisition, Resources. **Stavros Vologiannidis:** Conceptualization, Writing – review & editing, Project administration, Funding acquisition, Resources.

## Declaration of Competing Interest

The authors declare that they have no known competing financial interests or personal relationships that could have appeared to influence the work reported in this paper.

## Data Availability

StreetScouting dataset: A Street-Level Image dataset for finetuning and applying custom object detectors for urban feature detection. (Original data) (Zenodo).
